# Miniaturized spoof SPPs filter based on multiple resonators or 5G applications

**DOI:** 10.1038/s41598-021-01944-6

**Published:** 2021-11-19

**Authors:** Behnam Mazdouri, Mohammad Mahdi Honari, Rashid Mirzavand

**Affiliations:** 1 Intelligent Wireless Technology Laboratory, Electrical and Mechanical Engineering Department, University of Alberta, Edmonton, AB Canada; 2 Department of Electrical Engineering, University of Wisconsin-Madison, Madison, WI USA

**Keywords:** Engineering, Electrical and electronic engineering

## Abstract

This paper presents a novel and compact band-pass filter based on spoof surface plasmon polaritons (SSPPs) concept for 5G applications. In the first place, an SSPPs unit cell including L-shaped grooves and its equivalent circuit model are introduced. The obtained results from dispersion analysis shows that cut-off frequency of the cell can be considerably decreased thanks to its geometrical configuration. In the second place, a miniaturized SSPP transmission line (TL) consisting of the proposed unit cell with cut-off frequency of 29.5 GHz is designed. Two mode convertors have been employed for efficient connection between coplanar waveguides and SSPP TL. Moreover, a new method based on loading one unit cell of SSPP TL by stub resonators is proposed in order to block a specific frequency band. An equivalent circuit model for the cell with the resonators is proposed to predict rejected frequency range. Thereafter, as an example of our method, a SSPPs filter operating at 26.5–29.5 GHZ is designed by means of connecting stub resonators with different lengths to provide close resonance frequencies. The circuit model, full wave simulation, and measurement results are in a good agreement. The results of proposed groundless SSPP TL and filter structures are promising to make groundless 5G applications possible.

## Introduction

Over the years, the clients’ demands for wireless data services are increasing exponentially which are beyond what the current fourth mobile generation resources can afford^1^. The revolutionary fifth generation of telecommunication devices or 5G is expected to support such a high demanded capacity with higher data rates^2^. Furthermore, 5G brings an opportunity for such applications as massive MIMO^3^, Device-to-Device (D2D) or Machine-to-Machine (M2M) communications, and Internet of Things (IoT)^4^. 5G is considered as a millimetre wave technology with incredible data bandwidth, so that users will simultaneously send and receive nearly unfathomable amounts of data. Designing a filter with controllable bandwidth for 5G applications comes in useful due to optimum usage of spectrum resources. Spoof Surface Plasmon Polaritons (SSPPs) are good candidates for high-density integrated circuits and components at millimetre-wave and Terahertz frequency ranges thanks to their groundless structure and high field confinement^5^.

Surface plasmon polaritons (SPPs) are highly localized surface waves propagating parallel to the interface of metal and dielectrics and decaying exponentially in the normal direction to the metal. Since the metal has the similar property to plasma with a negative permittivity in the optical frequency region, SPPs only exists naturally in visible frequencies^6,7^. In virtue of perfect electric conductors of the metal at lower frequencies such as far infrared, terahertz and microwave bands, the plasmonic waves cannot be efficiently confined to the metal. To overcome the drawback at lower frequencies, the concept of spoof SPP (SSPP) metamaterials has been proposed to simulate propagation of plasmonic waves by corrugating a metal’s surface as a unit-cell and repeating it periodically cite^8,9^. Excellent physical characteristics of the so-called SSPPs can be manipulated by adjusting the geometrical parameters of the unit-cell conveniently.

Researchers have spurred a long-held interest to design electromagnetic structures based on SSPs concept in microwave and terahertz frequency regions. To generate SSPPs mode, SSPPs can be integrated through mode convertors to the conventional microwave or terahertz components, for instance, microstrip TL, coplanar wave guide (CPW-line), and substrate integrated waveguides (SIWs)^10,11^, or half-mode SIWs^12^. SSPPs has a very wide applications such as high efficiency SSPPs TL^13–17^, 3D SSPPs waveguides including one side L-shaped unit cells^18^, SSPPs antennas^19–21^, splitters^22^, and filters^23–29^. As a matter of fact, intrinsic slow-wave characteristic of SSPPs makes it very good candidate in designing of various microwave filters with different performances such as ultra-wideband^30^, band-stop^31–33^ or multiband filters^34^.

In previous researches in the field of designing filters with SSPPs, single-side and double-side SSP filters with several band-rejection performance have been proposed by etching complementary Split-ring resonators or SRRs^32^. A broadband band-pass filter composed of two asymmetrically broken corrugated strips coupled via the grooved strips with an embedded split ring resonator has been presented in^33^. In another work, a band rejection filter by using of embed multiple SRRs in SSPPs has been designed^35^. In addition to using SRRs and complementary SRRs in designing SSPPs filters, resonators with different configurations, for example, H-shaped resonators and complementary H-shaped slots have been utilized in a SSPP filter, so that complementary slots of the H-shaped periodic conductor units are embedded by multiple smaller H-shaped conductor strips in the middle^36^. In other proposed SSPPs filter configurations, a series of rectangular slots in the centre of metal strip^37^, a capacitive-coupled series SSPPs comprised of two H-shaped unit cells separated by a gap^38^, and two H-shaped cells in the center of non-periodic SSPPs fed by a CPW with defected ground planes^39^ have been employed to design SSPPs band-stop filters. Furthermore, metamaterial particles are introduced in the vicinity of the ultrathin corrugated metallic strip for controlling frequency rejections of SSPPs^40^. Moreover, To miniaturize SSPPs filters a multilayer SSPP band-pass filter based on the meanderline technology has been proposed^41^.

However, using multilayer structure, feeding SSPPs structures by SIWs or half mode SIWs, and utilizing coupled resonators in the vicinity of SSPPs TL (top or bottom coupling to SSPPs) make the designs either large and bulky or difficult for fabrication and integration to the other components. Also, in previous designs several resonating elements with exactly same dimensions were replicated to reject only a specific frequency region which results in longer size. Besides, some of the above-mentioned designs employed resonating elements with several geometric parameters which give rise to complicated design process. Here, firstly; we proposed and modelled a new 2D unit cell with increased inductive and capacitive parts compared with conventional H-shaped unit cell used in the previous designs to miniaturized size of a one layer two-side SSPPs TL. The SSPPs TL is fed by CPW lines through our proposed mode convertors. Secondly; we put forward a new simple method to reject undesired frequency regions by adding stub lines to inside of the designed unit cell. In fact, only one unit cell loaded by the mentioned stubs is adequate for rejection of a specific frequency region; therefore; our proposed structure can be used for connecting two one layer SPP structres in the left and right sides, for example, a SSPP TL and an SPP antenna in order to reject undesired frequency ranges. Furthermore, based on the proposed lumped element model, frequency response of the designed TL and band-rejected filter were predicted. Thereafter, a filter operating at 5G frequency band has been designed by manipulating rejection bands by means of stubs with different lengths inside the unit cell. The proposed structure based on this method is a compact and simple design which can be used in rejecting undesired frequency ranges.

## Results

### Proposed unit-cell and its equivalent circuit model

**Figure 1:**
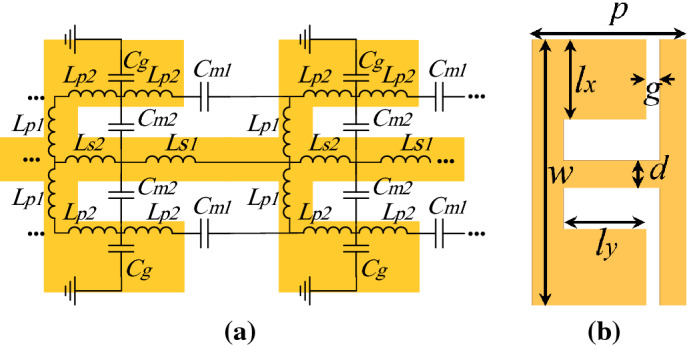
proposed unit cell (**a**) the schematic unit cell and (**b**) equivalent circuit model for two connected cells.

Figure 1a depicts the proposed unit cell consisting of a corrugated metallic surface with double-side L-shaped grooves. Characteristics of the SSPPs unit cell are controlled by its geometrical parameters, which are the gap between grooves *g*, the width of cell *w*, L-shaped dimensions including width of $l_x$ and length of $l_y$, width of central conductor d , and period of *p*. By referring to the ground plane at infinity in SSPPs, the equivalent circuit model for the unit cell is presented in Fig. 1b. To model electric field in the gap between L-shaped grooves and next cell, the capacitances $C_{m1}$ connects the two cells. Also, $C_{m2}$ is the capacitance between L-shaped grooves and central conductor in one unit cell, and $C_g$ is the capacitance between the cell to a isolated ground plane in infinity. The equivalent inductors $L_{S1}$, $L_{S2}$, $L_{p1}$, $L_{p2}$ model the surface current at the SSPPs cell. Inductive and capacitive components can be calculated analytically^42,43^.

**Figure 2:**
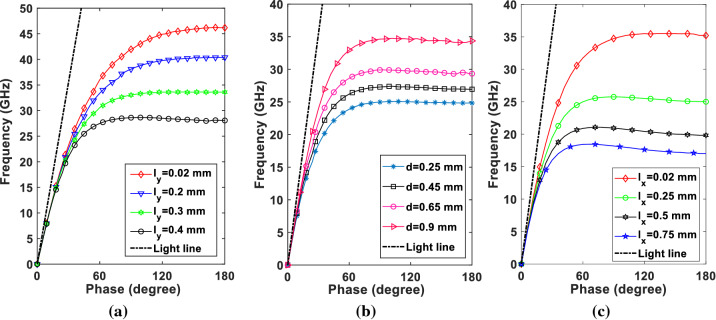
Dispersion diagrams showing the effect of geometrical parameters variation. (**a**) Varying $l_y$ by keeping $W=2.43$, $l_x=0.5, p=0.7, d=0.54$, and $l_y+g=0.48$. (**b**) Varying *d* by keeping $W=2.34, l_x=0.5, p=0.7, l_y=0.43$, and $g=0.05$. (**c**) Varying $l_x$ by keeping , $W-l_x=0.4, l_y=0.43, p=0.7, d=0.54, g=0.05$ (all dimensions are in mm). CST microwave studio 2020 (https://www.3ds.com/products-services/simulia/products/cst-studio-suite/) and MATLAB 2021 (https://www.mathworks.com/products/matlab.html) were used to generate this figure.

To study the SSPP behaviour of the proposed unit cell in periodic mode, Eignmode analysis has been performed to calculate dispersion diagram of TM polarized wave in the introduced structure. In our analysis, we assume that the corrugated metal with L-shaped grooves is printed on a 0.2 mm thick dielectric substrate RO4003 with dielectric constant of 3.55 and loss tangent of 0.002. The conductor is chosen as annealed copper with film thickness of 18 $\upmu$m. The dispersion curve of the fundamental mode for the proposed unit cell are shown in Fig. 2a–c. Figure 2a plots the dispersion curves of SSPP cell for various lengths of $l_y$. The other parameters are set as $p=0.7$ mm, $l_x=0.5$ mm, $d=0.54$ mm, $W=2.34$ mm, and $l_y+g=0.48$ mm. According to Fig. 2a, increasing the length of $l_y$ from 0.02 to 0.4 mm decreases the cut-off frequency of the unit cell from approximately 47–28 GHz. This means nearly 41% decreasing in cut-off frequency compared with conventional H-shaped unit cell (for $l_y=0$). Figure 2b presents dispersion curves for various d values while other parameters have constant values: $p=0.7$ mm, $g=0.05$ mm, $l_x=0.5$ mm, $l_y=0.4$ mm, and $W=2.34$ mm. Figure 2c compares the cut-off frequencies of the unit cell for different values of $l_x$ while other parameters are constant, $p=0.7$ mm, $g=0.05$ mm, $d=0.54$ mm, $l_y=0.4$ mm, and $W-l_x=0.4$ mm . It is obvious from the Fig. 2c that lower cut-off frequency can be achieved by increasing the parameter $l_x$.

### Proposed SSPPs TL

**Figure 3:**
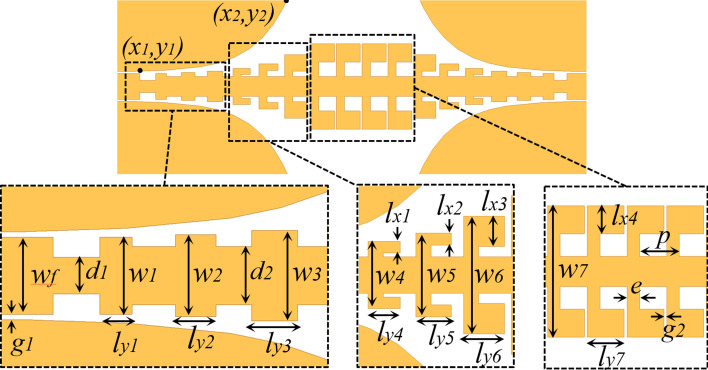
Schematic picture of the proposed transition with gradient L-shaped grooves and two flaring grounds. Detailed dimensions are determined as := $w_1=0.715, w_2=0.76, w_3=0.84, w_4=1, w_5=1.24, w_6=1.74, w_7=2.34, l_{y1}=0.3, l_{y2}=0.38, l_{y}3=0.42, l_{y4}=0.5, l_{y5}=0.52, l_{y6}=0.6, l_{y7}=0.65, l_{x1}=0.17, l_{x2}=0.18, l_{x3}=0.45, l_{x4}=0.5, d=0.34, d_2=0.54, g_2=0.05, p=0.7, e=0.22$ (all dimensions are in *mm*). CST microwave studio 2020 (https://www.3ds.com/products-services/simulia/products/cst-studio-suite/) was used to generate this figure.

In this section, we intend to present a miniaturized SSPPs TL comprised of the unit cell with L-shaped grooves opearating at 28 GHz for 5G applications. For this purpose, we designed a unit cell with cut-off frequency of 29.5 GHz, and the obtained dimensions are: $d=0.54, W=2.34, l_x=0.5, p=0.7, l_y=0.43$, and $g=0.05$ (all dimensions are in mm). In order to apply SSPP modes in microwave and millimetre wave circuits, we need to design mode convertors according to the introduced unit cell. Figure 3 demonstrates the suggested mode convertor so as to connect the conventional CPW line to the SSPPs TL. In transition part, to have an efficient conversion from the quasi TEM mode of the CPW line to SSPP TM mode, the width of unit cells are gradually increased and altered to L-shaped configuration. Additionally, the flaring grounds with exponential outline are employed to smoothly omit the metallic ground planes in both sides. According to the Fig. 3, the exponential curve of ground planes can be determined by starting point ($x_1$, $y_1$) and ending point ($x_2$, $y_2$), and it can be optimized to have a good matching between the CPW line and the SSPP TL.

The parameters $W_f$ and $g_1$ shown in Fig. 3 are chosen 0.715 mm and 0.05 mm respectively to get 50-ohm impedance for the CPW line. The detailed dimensions of other parameters were given in Fig. 3.

**Figure 4:**
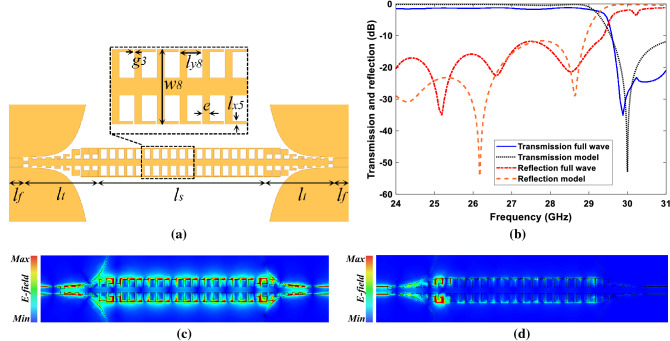
The proposed SSPPs TL. (**a**) Top view of the proposed SSPPs TL. Detailed dimensions are determined as: $g3= 0.05, w8=2.43, e=0.225, l_x5=0.1, l_y8=0.43$ (all dimensions are in *mm*). (**b**) Transmission and reflection coefficients of the lossless SSPPs TL from equivalent circuit model compared with full wave simulation. (**c**) Simulated electric field distributions of the proposed at the frequency of 28 GHz and (**d**) 30 GHz. CST microwave studio 2020 (https://www.3ds.com/products-services/simulia/products/cst-studio-suite/) and MATLAB 2021 (https://www.mathworks.com/products/matlab.html) were used to generate this figure.

Figure 4a illustrates our proposed SSPPs TL on a 0.2 mm thick dielectric substrate RO4003 with dielectric constant of 3.55 and loss tangent of 0.002. The line consists of three concatenated parts, which are two 50-ohm CPW line on the left and right sides with length of $l_f$, two mode convertors on the both sides with length of $l_t$, and groundless SSPPs TL with length of $l_s$. The length of $l_s$ was set to 13.4 mm equal to 1.25 of wave-length at 28 GHz in free space. The designed 50-ohm CPW line and mode convertors, dimensions were preceded in Fig. 3, have been utilized to attain SSPP TM mode for feeding SSPPs part. To predict transmission and reflection coefficients of the SSPPs line, the suggested circuit model in Fig. 1b comes in handy to analytically model corrugated metals with L-shaped grooves with different width from $w_4$ to $w_8$.Furthermore, for the rest of corrugations with width of $d_1$, $d_2$, and $w_1$ to $w_4$, CPW lines with curvy ground planes were considered for the modelling. For instance, the corrugation with width of $w_2$ can be modelled as a CPW line with a gap between maximum and minimum distance of central conductor to its curvy ground. A similar approach reported in previous studies has been employed as a starting point for modelling of the SSPP TL^42,43^. For the 50 ohm CPW line ($W_f$) and a part of transition ($W_1$, $d_1$, $W_2$, $d_2$, and $W_3$), eight CPW lines with different distances to the ground planes on the sides were used. The rest of transition part was modelled like the SSPP line. Then the model with this approach was optimized to have similar S-parameters obtained from the full wave simulation. According to the Fig. 1b, values of the circuit components for the proposed one unit cell of the SSPP TL are: $L_{p1}= 0.05$ nH, $L_{p2}= 0.09$ nH, $L_{s2}= 0.02$ nH, $L_{s1}= 0.17$ nH, $C_{m2}=1$ fF, $C_{g}=34$ fF, $C_{m1}=300$ fF. Figure 4b presents the transmission and reflection results corresponding to proposed model and full wave simulations. From Fig. 4b, it is conspicuous that the obtained results are in good agreement.

In order to give a physical insight to the operating mechanism of the SSPPs line at the frequencies upper and lower than the cut-off frequency of 29.5 GHz, the simulated electric field distribution at 28 GHz and 30 GHz were shown in Fig. 4c, d, respectively. As shown in Fig. 4c, the electromagnetic wave propagates at our desired frequency of 28 GHz, but at 30 GHz it will be blocked at the beginning of SSPPs line by the designed unit cell.

**Figure 5:**
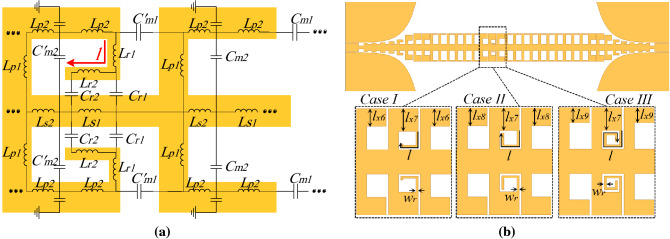
Connection of stub resonators to the SSPP line. (**a**) The unit cell with connected stub resonator and its circuit model. (**b**) A SSPPs TL with a unit cell loaded by stub resonators in three cases, $l=0.73$ in case I, $l=0.83$ in case II, $l=1$ in case III. CST microwave studio 2020 (https://www.3ds.com/products-services/simulia/products/cst-studio-suite/) was used to generate this figure.

### Band-Rejected SSPPs TL with one unit cell loaded by resonating elements

Since we intend to reject a specific frequency range lower than the cut-off frequency of the unit cell, we add resonating elements to the circuit model shown in Fig. 1b. A certain frequency range can be rejected by designing a resonating element which resonates at that frequency. Utilizing this element means adding inductive and capacitive components to the circuit model. Regarding the geometrical feature of L-shaped grooves in the proposed unit cell, it is very simple to increase coupling between cells. Figure 5a presents our proposed stub resonator connected to the cell. The equivalent circuit model of the unit cell loaded by the resonating element was shown in Fig. 5a. According to Fig. 5a, connecting a stub with length of *l* to the L-shaped grooves either easily increase coupling between two cells or add inductive component to the model. In the model $L_{r1}$, $L_{r2}$, $C_{r1}$, and $C_{r2}$ represent inductive and capacitive components related to the resonator. Furthermore, in the cell with resonator, the capacitive components $C_{m1}$ and $C_{m2}$ have been replaced by the $C_{m1}$ and $C_{m2}$.

Figure 5b demonstrates the designed SSPPs line including two stub resonators connected to the L-shaped grooves of one unit cell in three cases. From case I to Case III the length of stubs have been increased ($l=0.73$ in case I, $l=0.83$ in case II, $l=1$ in case III) with constant $w_r$ and $l_{x7}$ to achieve different resonant frequencies. According to this figure, the length of L-shaped grooves in the next cells $l_{x6}$, $l_{x8}$, and $l_{x9}$ can be tuned to have a better impedance matching to the CPW. For example, the dimensions in Fig. 5b are as: $w_r=0.05$, $l_{x6}=0.4$, $l_{x7}=0.5$, $l_{x8}=0.4$, $l_{x9}=0.3$ for the designed SSPPs TL in Fig. 4a.

**Figure 6:**
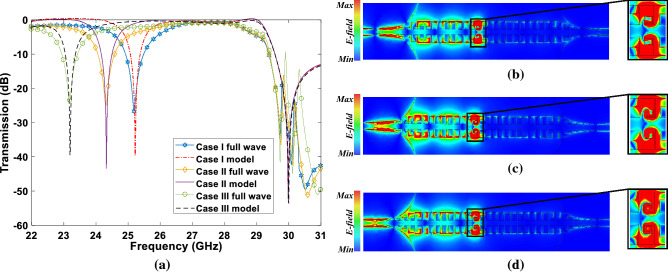
Results of SSPPs TL loaded by stub resonator. (**a**) Transmission coefficient obtained from the proposed model compared with the full wave simulations. Electric field distribution of the filter at (**b**) 25.2 GHz, (**c**) 24.3, (**d**) 23.2 GHz. Keysight 2021 (https://www.cmc.ca/keysight-pathwave-cmc-00200-00872/) software and MATLAB 2021 (https://www.mathworks.com/products/matlab.html) were used to generate this figure.

The three cases in Fig. 5b were modelled by using of the equivalent circuit in Fig. 5a. Figure 6a shows the transmission coefficient obtained from the analytical model compared with the full wave simulations. As can be seen in this figure, the obtained resonant frequencies for the connected stub in the case I to case III are 25.2 GHz, 24.3 GHz, and 23.2 GHz respectively. It is worth mentioning that existence of the transmission peaks at the frequencies above the cutoff frequency is because of back and forth transformation of magneto inductive waves to SSPP. In other words, the two stubs on the two sides and L-shaped coupled unit cells cause this effect. Similar results have been reported in the previous studies^44^.

To have a better physical sense of frequency rejection by the proposed method, Fig. 6b–d shows the electric field distribution at the resonant frequency of the resonators 25.2 GHz, 24.3 GHz, and 23.2 GHz, respectively. In Fig. 6b. It is obvious that the electromagnetic wave has been blocked at the mentioned frequencies by the connected stubs.

**Figure 7:**
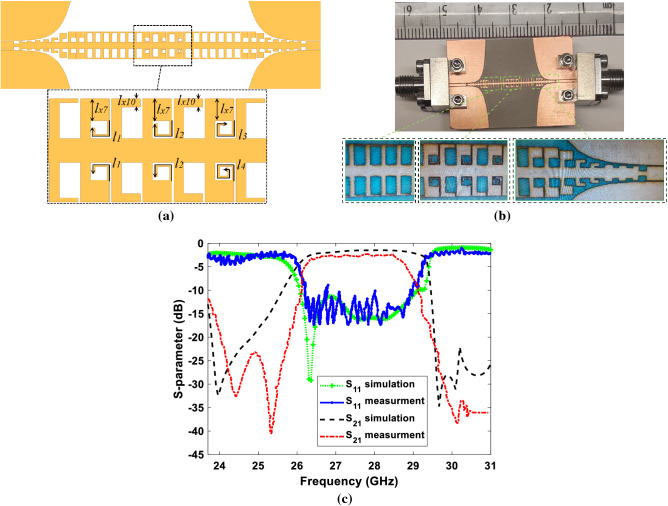
Proposed SSPPs filter (**a**) Schematic picture of the designed SSPPs filter consists of stub resonators with different lengths. (**b**) The fabricated filter. (**c**) Simulated scattering Ansys 2021 (https://www.cmc.ca/ansys-campus-solutions-cmc-00200-04847/) parameters compared to the measured ones.

**Figure 8:**
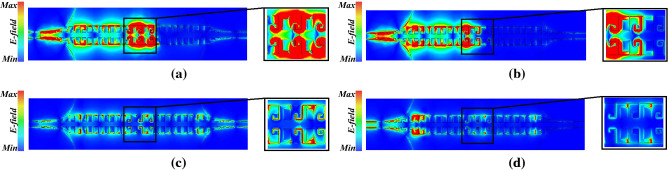
Electric field distribution of the filter at (**a**) 23.6 GHz, (**b**) 24.5 GHz, (**c**) 28 GHz, (**d**) 30 GHz. CST microwave studio 2020 (https://www.3ds.com/products-services/simulia/products/cst-studio-suite/) was used to generate this figure.

### Proposed SSPPs filter with multiple cells loaded by resonating elements

Regarding the results obtained so far, we aim to manipulate rejection bands for designing a SSPPs filter. Based on the aforementioned method, only one unit cell loaded by the stub resonator is enough for rejecting a specific frequency range. For manipulation of the stop-band in a SSPPs filter, we propose utilizing multiple stub resonators with different lengths simultaneously. Figure 7a reveals the proposed filter including three unit cells loaded by resonating stubs with different lengths $l_1$ to $l_4$. The length of stubs $l_1$ to $l_4$ can be determined analytically so as to resonate at close frequencies. Designing stub resonators with close resonances results in rejection of a wider frequency range as wide as required for different applications. Figure 7b shows the fabricated band-pass filter operating at 26.5–29.5 GHz, which the dimensions are as:$l_1=0.73, l_2=0.83, l_3=1, {l_4=1.2}, l_{x7}=0.5, l_x10=0.2$ (all units are in *mm*). The upper cut-off frequency has been dedicated by the dimension of the unit cell at 29.5 GHz which has been discussed in previous sections. Figure 7c illustrates the scattering parameters of the fabricated filter in comparison with the simulation ones.

Figure 8 shows the electric field distribution of the filter at different frequencies to give a physical insight into the operating mechanism of the proposed filter in stop-band and pass-band. Figure 8a, b shows electric field distribution blocked by the proposed stub resonators at 23.6 GHz and 24.5 GHz, respectively. It is conspicuous that at 23.6 GHz longer stubs play key role in rejection of input wave while at 24.5 GHz shorter stubs are responsible for rejecting the input wave.

## Discussion

In summary, we have proposed a new unit cell with L-shaped grooves to design either a miniaturized SSPPs TL or a novel filter with simple design procedure. Dispersion analysis of the unit cell indicates that it comes in useful to considerably reduce size of the SSPPs structures. The conventional CPW has been employed to excite the miniaturized SSPPs TL through two novel transition sections for smoothly converting the CPW mode to the SSPP mode. Moreover, a new method is presented to reject a specific frequency range by means of loading merely one unit cell of the SSPP TL with stub resonators. In fact, geometrical feature of the unit cell provides a potential to simply increase coupling between cells by connecting it to the stub resonators. Thereafter, by using of this method, a SSPPs filter operating at 26.5–29.5 GHz is suggested. Upper cut-off frequency of the filter can be dedicated by dimension of the cell while lower cut-off frequency can be determined by manipulating of physical dimensions of the added stub resonators. The performance of the filter has been analysed using our proposed equivalent circuit models for the unit cell and stub resonator. Good agreement has been found between results from the analytical models, full wave simulation, and measurements. Finally, the proposed SSPP TL and filter are compact and easy for fabrication and integration. Such advantages comes in handy to make groundless 5G devices possible.
